# The Effect of Borage Seed Oil (*Borago officinalis* L.) and Matcha Tea Powder (*Camellia sinensis* L.) on the Physicochemical Properties, Oxidative Stability, Color, and Tenderness of Vacuum-Packed Lamb Meatloaf During Storage

**DOI:** 10.3390/foods14203500

**Published:** 2025-10-14

**Authors:** Agnieszka Latoch, Justyna Libera, Nilgün Öncül, Murat Metli

**Affiliations:** 1 Department of Animal Food Technology, University of Life Sciences in Lublin, Skromna 8 St., 20-704 Lublin, Poland; agnieszka.latoch@up.lublin.pl; 2 Department Engineering and Cereal Technology, University of Life Sciences in Lublin, Skromna 8 St., 20-704 Lublin, Poland; 3 Department of Nutrition and Dietetics, Muğla Sıtkı Koçman University, Fethiye 48300, Muğla, Türkiye; nilgunoncul@mu.edu.tr; 4 Department of Food Hygiene and Technology, Muğla Sıtkı Koçman University, Milas 48200, Muğla, Türkiye; muratmetli@mu.edu.tr

**Keywords:** lamb meatloaf, storage time, antioxidants, oxidative stability, matcha

## Abstract

In this study, the effects of borage seed oil (BO) and matcha tea (MT) on the physicochemical properties, oxidative stability, color, and tenderness of vacuum-packed lamb meatloaf during 14 days of refrigerated storage were evaluated. No significant effect of the plant additives was observed on cooking yield (66%) or pH (≈5.95). Samples with added oil had a higher fat content, which contributed to hydrolytic and oxidative changes that were already evident immediately after production. The addition of MT, as well as the combined use of both BO and MT, effectively limited these changes. After 14 days, TBARS values in these samples decreased by up to 80% compared to the control. Neither BO nor MT affected lightness (L*) or yellowness (b*), whereas matcha, being naturally green, reduced redness (a*) by about 50%. However, no significant differences in total color (ΔE* < 4) were observed between control and experimental samples. The addition of BO also had a significant effect on the texture of meatloaves, which showed a 15% reduction in shear force after 14 days of storage. In conclusion, the combined application of BO and MT effectively limited fat oxidation while maintaining desirable color, tenderness, and overall physicochemical quality of meatloaves comparable to the control.

## 1. Introduction

Lamb meat is highly valued in global markets not only for its palatability but also for its rich nutritional profile. It is a source of high-quality protein and essential amino acids, as well as heme iron and B vitamins [1]. Being meat from a young animal, lamb contains a favorable balance of saturated and unsaturated fatty acids, including conjugated linoleic acid (CLA) and omega-3 polyunsaturated fatty acids (PUFAs), which have been associated with cardiovascular and metabolic health benefits [2,3]. The composition of muscle fibers in lamb meat affects its culinary quality attributes. Lamb meat exhibits a higher proportion of oxidative fibers, which significantly enhances both the sensory and nutritional properties of the meat by modulating lipid metabolism, amino acid availability, and flavor development [4]. Lamb meat products, especially from native breeds [5], represent a suitable raw material for processed meat products, particularly when enriched with plant-based sources of bioactive compounds during processing.

Considering the current trend toward clean-label foods, ongoing research focuses on identifying and characterizing plant-derived antioxidant compounds, such as plant oils rich in polyunsaturated fatty acids (PUFAs) and teas abundant in catechins [6]. Borage (*Borago officinalis* L.) is an annual herb primarily cultivated for medicinal purposes. Oil is produced from its seeds [7], which is rich in bioactive fatty acids, including gamma-linolenic acid (26–38%), linoleic acid (35–38%), and oleic acid (16–20%), along with smaller amounts of palmitic acid (10–11%), stearic acid (3.5–4.5%), eicosenoic acid (3.5–5.5%), and erucic acid (1.5–3.5%) [8]. Borage seeds also contain high levels of flavonoids with anti-inflammatory and antioxidant properties; tannins with antibacterial activity; saponins, which enhance nutrient absorption from the intestines into the bloodstream, stimulate the secretion of gastric, bile, and intestinal juices, may influence cholesterol levels, and promote fat digestion; and mineral salts such as iron, zinc, magnesium, calcium, copper, manganese, silicon, and potassium. Due to the high content of these bioactive compounds, borage seed oil is used in the treatment of various disorders, including multiple sclerosis, diabetes, cardiovascular diseases, arthritis, and eczema [9]. Although borage seed oil is edible, it is rarely used as a culinary oil [10] and is more commonly applied in cosmetology and medicine. Meanwhile, in scientific research, water extracts of borage are most frequently used [11,12,13,14,15,16].

Matcha (*Camellia sinensis* L.) is a finely powdered green tea produced from fresh tea leaves grown in the shade, steamed or hot-air dried, and processed by grinding [17]. Matcha retains nearly all the nutritional and health-promoting properties of green tea, and its powdered form broadens its potential applications. Green tea is widely recognized for its antioxidant properties. The beneficial effects of green tea extracts are attributed to the presence of polyphenols, polysaccharides, vitamins B, C, and E, and amino acids [18]. The most important group of bioactive compounds are polyphenols (phenolic acids, stilbenes, flavonoids, and lignans), comprising approximately 30% of the dry leaf mass [19]. Among the flavonoids (catechins) are epicatechin, epicatechin gallate, epigallocatechin, and epigallocatechin gallate, with the latter being the most abundant and biologically active component of matcha [20,21]. Its characteristic chemical composition and unique flavor distinguish it from other teas [22]. Furthermore, green tea exhibits notable health-promoting effects, including anticancer activity, cardiovascular protection, anti-obesity and anti-diabetic effects, oral and bone health benefits, and enhancement of burn and wound healing [23,24,25].

The combined use of both plant-based additives—borage seed oil (BO) and matcha tea (MT)—may exert a synergistic effect, which motivated the present study. The aim was to evaluate the impact of these natural additives—borage seed oil (*Borago officinalis* L.) and matcha tea (*Camellia sinensis* L.)—on the physicochemical properties, oxidative stability, color, and tenderness of vacuum-packed lamb meatloaf during 14 days of storage at 4 °C.

## 2. Materials and Methods

### 2.1. Materials

The research material consisted of lamb meatloaves. The meat was obtained from lambs of the native Polish Świniarka breed, which were raised according to organic farming standards [26] in the Lublin region, Poland. From the second week of life, the lambs received meadow hay and crushed oats along with their mother’s milk, and from the fifth week, hydrated pulp. At 8 weeks of age, the lambs had an average body weight of 15.7 kg. On day 90, they were weaned and fed exclusively with meadow hay, supplemented with fresh forage (a mixture of grasses and alfalfa) and crushed oats, modified every two weeks. At four months of age, they grazed on xerothermic grasslands within a Natura 2000 protected area. Five lambs were selected for the study. Animal care complied with the Guide for the Care and Use of Laboratory Animals [27], Directive 2010/63/EU [28], and Directive 1998/58/EC [29]. At seven months of age, the lambs had an average body weight of 29 kg and were slaughtered based on farming efficiency and meat processing requirements. Transport and slaughter were carried out by a certified company at a small abattoir supervised by the Veterinary Inspection, in accordance with EU regulations. Carcasses were held at 10–15 °C for 4 h and subsequently chilled at 3–4 °C for 20 h. Meat from the deboned carcasses and lamb legs was vacuum-packed and transported at 4–6 °C for use in the present experiment.

The plant-based additives originated from the southwestern region of Türkiye. Borage seed oil (*Borago officinalis* L.) 100% pure (Polente Natural Hodan, Istanbul, Türkiye), cold-pressed, was purchased from a local pharmacy in Mugla (Türkiye). A product with a high content of polyunsaturated fatty acids was selected for the experiment. According to the manufacturer’s declaration, 100 g of the oil contains 27 g of monounsaturated fatty acids, including 18 g of oleic acid (C18:1), and 57 g of polyunsaturated fatty acids, including 37 g of linoleic acid (C18:2) and 20 g of gamma-linolenic acid (C18:3n-6). Matcha tea 100% (*Camellia sinensis* L.) (Şölen Çay Tic. Ltd. Şti., Sürmene, Turkey) was purchased from an organic food store in Fethiye (Turkey).

### 2.2. Lamb Meatloaf Preparation

The meat was ground using a meat grinder (KU2-3EK, Mesko-AGD, Skarżysko-Kamienna, Poland) with an 8 mm plate. All ingredients (Table 1) were mixed in a universal mixer (KU2-3EK, Mesko-AGD, Skarżysko-Kamienna, Poland) equipped with an R4 paddle (100 rpm for 3 min). Plant additives were incorporated into the mixture during mixing, resulting in three experimental lamb meatloaves: BO + MT—with borage seed oil (0.4 g/kg of meat) and matcha powder (0.05 g/kg of meat), BO—with borage seed oil (0.4 g/kg of meat), MT—with matcha powder (0.05 g/kg of meat), and CON—control meatloaf was prepared without plant additives. The concentrations of the plant additives were selected based on our own pilot studies, the results of which have not yet been published. Each portion of the mixture (approximately 300 ± 10 g) was placed in aluminum baking trays and baked in a convection oven (XF 135 UNOX S.p.A. Padova, Italy) at 160 °C until the internal temperature at the geometric center reached 72 °C. After cooling, the samples were vacuum-packed in polyethylene bags and stored in a refrigerator at 4 °C. The product was analyzed at three storage times: 24 h, 7 days, and 14 days. The experiment was conducted twice, one week apart, under identical conditions. Analyses were performed on replicates.

### 2.3. Lamb Meatloaf Preparation for Analysis

The samples were ground twice using a 3 mm mesh in a grinder (Zelmer, Rzeszów, Poland). The ground bulk was thoroughly mixed, and then samples were collected for all assays.

### 2.4. Physicochemical Properties of Vacuum-Packed Lamb Meatloaf

Cooking yield was calculated based on the difference in weight before and after heat treatment. Moisture content was determined using ISO method [30]. Fat content was determined during the FFA test (described in Section 2.5). For pH measurement, a digital CPC-501 pH meter (Elmetron, Zabrze, Poland) equipped with a temperature sensor and ERH-111 pH electrode (Hydromet, Gliwice, Poland) was used. The pH measurement was performed on a homogenate of the meatloaf and distilled water (1:5 ratio) prepared just before the measurement [31].

### 2.5. Oxidative Stability and Hydrolysis of Vacuum-Packed Lamb Meatloaf

The content of free fatty acids (FFA) in meatloaf was determined according to the method described by Koniecko [32] with modifications by Malik and Sharma [33]. A homogenate prepared from 10 g of minced meatloaf, 60 mL of chloroform, and 10 g of anhydrous sodium sulfate was filtered through Whatman No. 1 filter paper. Twenty mL of the filtrate were dried in an oven (SPU-200, ZUT Colector, Cracow, Poland) to determine the fat content. The remaining filtrate was titrated with 0.1 M ethanolic potassium hydroxide solution against 0.2% phenolphthalein until a light pink color appeared. The quantity of potassium hydroxide consumed during the titration was recorded. FFA as percentage of oleic acid was calculated as follows:

FFA (% oleic acid) = (2.82 ⋅ mL 0.1 N potassium hydroxide)/mass of fat (g)

The peroxide value was determined according to the procedure described by Koniecko [32], with Wagh et al. [34] modifications. A 5 g portion of the sample was homogenized with 30 mL of chloroform in the presence of anhydrous sodium sulfate for 2 min. The mixture was filtered through Whatman No. 1 filter paper, and a 25 mL aliquot of the filtrate was transferred into a 250 mL conical flask. Subsequently, 30 mL of glacial acetic acid and 2 mL of saturated potassium iodide solution were added, and the mixture was allowed to stand for 2 min with occasional swirling. Thereafter, 100 mL of distilled water and 2 mL of freshly prepared 1% starch solution were added. The contents were titrated immediately with 0.1 N sodium thiosulfate solution until the end point was reached (disappearance of the color in the non-aqueous layer). The PV as milliequivalents of peroxide per kg was calculated as follows:

PV (mEq/kg) = (10 ⋅ mL 0.1 N sodium thiosulfate)/sample weight (g)

Lipid oxidation (TBARS) was investigated using 2-thiobarbituric acid (TBA), according to Pikul et al. [35] with slight modifications. Four grams of meatloaf were homogenized with 12 mL of cold perchloric acid (4%) and 200 μL of an alcohol solution (0.01%) of BHT at 1300 g for 2 min using a homogenizer (T25 Basic Ultra-Turrax, IKA, Staufen, Germany). Then, it was filtered through Whatman No.1 filter paper. Subsequently, 650 μL of filtrate and TBA solution (0.02 mol/L in distilled water) were mixed. A mixture of 650 μL of 4% cold perchloric acid and 650 μL of TBA solution was used as the reference sample. The heating part of the experiment was performed for 20 min at 100 °C. Then, spectrophotometrically (U-5100 UV-Vis, Hitachi High Technologies America Inc., Schaumburg, IL, USA) the absorbance at 532 nm was measured. The TBARS value as mg malondialdehyde per kg was calculated as follows:

TBARS (mg MDA/kg) = 5.5 ∙ Absorbance at 532 nm

### 2.6. Color Parameters CIE L*a*b* of Vacuum-Packed Lamb Meatloaf

The color of lamb meatloaves was measured using an spectrophotometer (Color 8200, X-Rite Inc., Grand Rapids, MI, USA) with a port (Ø 12 mm) and the D65 illuminant, and standard observer 10. The device was calibrated, and color measurements were performed at room temperature at fifteen different locations of sample. The color was expressed in the CIE L*a*b* space, where L*—lightness, a*—redness, and b*—yellowness [36].

The total color difference (ΔE*) was calculated based on the ΔL*, Δa*, and Δb* results for each sample with the plant additive compared to the control sample (CON), for three time intervals of 1, 7, and 14 days. The formula used for the calculation was:

ΔE* = [(ΔL*)^2^ + (Δa*)^2^ + (Δb*)^2^]^1/2^

ΔE* values below 2 indicate a color difference that is barely perceptible. Values in the range of 2–3.5 suggest that an inexperienced observer would notice the difference, while values above 3.5 indicate a clearly perceptible and significant color difference [36].

### 2.7. Tenderness of Vacuum-Packed Lamb Meatloaf

Textural characteristics were carried out using Warner–Bratzler Shear Force test [37]. The Warner–Bratzler ‘V’ slot blade was attached to the universal testing machine TA.XT2 (Stable Micro System Corporation, Godalming, UK) and the shearing speed was 2 mm/s. The load cell capacity used in this study was 50 kg. The sample diameter was about 10 mm. To assess the tenderness of the meatloaf samples two parameters were measured: the maximum shear force (N) and work of shearing (N∙mm)—the total shear work necessary to cut through the sample. At least ten technical replicates were used for each sample.

### 2.8. Statistical Analysis

All calculations and comparisons were performed using Statistica version 13.3 (Dell, Inc., Round Rock, TX, USA). Data were analyzed using ANOVA for factorial designs. A two-way ANOVA was applied for statistical comparisons between the treatment (type of plant additive), storage time, and the measured parameters. Tukey’s test was used for post hoc comparisons at a 95% confidence level (α = 0.05). Differences were considered statistically significant at *p* > 0.05. Results are presented as mean ± standard deviation. Principal Component Analysis (PCA) was used to evaluate and classify the main variables of all samples. The experiment was conducted twice, with analyses performed in at least three replicates.

## 3. Results

### 3.1. Physicochemical Properties of Vacuum-Packed Lamb Meatloaf

In the cooking performance assessment, the results indicated that neither the 0.5% addition of matcha tea powder nor the 4% addition of borage seed oil had an effect on cooking yield or moisture content (Table 2). However, the addition of 4% oil clearly increased the fat content in the samples containing it. The pH values of all lamb meatloaves remained stable (ranging from 5.92 to 5.98) and did not change during 14 days of refrigerated storage in vacuum-packed conditions.

### 3.2. Oxidative Stability and Hydrolysis of Vacuum-Packed Lamb Meatloaf

Hydrolytic changes contribute to the deterioration of the shelf life of meat products, which is why selecting appropriately high-quality raw meat is important (in this study, organically raised lamb was used). Furthermore, the use of additives with antioxidant potential (matcha), along with proper storage conditions (exclusion of air and light, and low temperature), limits the development of hydrolytic and oxidative processes, thereby helping to preserve the quality of the meat product [38]. The content of free fatty acids (FFA) reflects the extent of fat molecule decomposition, and both the addition of plant-based ingredients and storage time influenced the amount of oleic acid measured by the FFA method. In products stored for 14 days, FFA increased by approximately 33% (from 1.8% to 2.7% oleic acid) (Table 3). Matcha (MT) alone significantly reduced (*p* < 0.05) the FFA concentration in fresh meatloaf (1.5%) compared to the control sample (1.8% oleic acid). However, MT combined with BO was less effective at inhibiting fat hydrolysis, and the FFA content in the BO + MT sample was approximately one-third higher than in the MT-only sample.

Peroxide value (PV) is an indicator of the formation of primary lipid oxidation products and reflects the extent of fat oxidation in meat. PV analysis is based on determining the amount of iodine released from potassium iodide under the influence of peroxides present in the tested fat. In this study, changes in PV were observed (Table 3). Overall, PV in stored meatloaves increased by approximately 13% (from 7 meq O_2_/kg on day 1 to 8 meq O_2_/kg on day 14). Initially, PV decreased by 0.6 meq O_2_/kg after 7 days of storage, followed by a significant (*p* < 0.05) increase, reaching an average of 8.3 meq O_2_/kg in the oil-containing samples (BO and BO + MT). The smallest changes (3%) were observed in the control sample, which already had the highest initial peroxide content (7.1 meq O_2_/kg) on day 1. In meatloaf with added matcha tea (MT), changes in PV were more subtle (6.8 meq O_2_/kg on day 1, 6.6 meq O_2_/kg on day 7, and 8 meq O_2_/kg on day 14), indicating that the addition of MT-only effectively limited peroxide formation during the 7-day storage period.

TBARS represent a group of various chemical compounds, including malondialdehyde. When these compounds react with 2-thiobarbituric acid (TBA) at elevated temperatures, colored complexes are formed. TBARS are quantified by measuring the intensity of these complexes; the greater the amount of lipid-derived aldehydes in the sample, the more intense the color. TBA reacts particularly readily with substances containing carbonyl groups. The maximum consumer-acceptable TBARS level has been established at 2 mg per 1 kg of product [26], and this threshold was not exceeded in the present study (Table 3). The addition of matcha, which is rich in antioxidants significantly (*p* < 0.05) stabilized lipid oxidation in meatloaf. In samples treated with BO and the control, TBARS levels increased with prolonged storage. In contrast, in meatloaves containing matcha (MT and BO + MT), TBARS values after 7 and 14 days of storage remained as low as in the freshly prepared product, approximately 0.5–0.7 mg/kg. For comparison, the control sample reached 2 mg/kg. These results confirm the effectiveness of tea, including matcha, as a natural antioxidant in meat product technology.

### 3.3. Color Parameters CIE L*a*b* of Vacuum-Packed Lamb Meatloaf

Analysis of the results from vacuum-packed samples stored at refrigeration for 24 h, 7 days, and 14 days showed no significant (*p* < 0.05) effect of the plant-based additives on the lightness (L*) and yellowness (b*) parameters (Table 4). Although not statistically significant (*p* > 0.05), the BO sample was the brightest (highest L* value). In each of the storage periods, this sample had slightly higher L* values than the others. Conversely, the addition of MT caused a darkening of the color. This sample exhibited lower, but not statistically significant (*p* > 0.05), L* values across all storage periods. The addition of plant-based ingredients, particularly MT, reduced (*p* < 0.05) the intensity of red color (a*) compared to the control (CON). With extended storage time, a* values decreased in both CON and plant-supplemented samples. However, the most pronounced (*p* < 0.05) decline in a* over time was observed in the BO sample. In the other samples, a significant decrease occurred only after 14 days. Analysis of the b* parameter indicated that plant additives affected yellowness compared to CON. Higher values correspond to greater yellow color saturation. The highest values were observed in the BO + MT sample, while the lowest were recorded in CON. The addition of both plant ingredients caused a significant (*p* < 0.05) increase in yellow saturation throughout the storage period compared to CON. For the MT sample, storage time significantly reduced b*, whereas in the other samples, a decline in yellowness was also observed, but it was not significant (*p* > 0.05). Overall, no clear trend in b* changes over time was observed, although the highest b* values occurred in all samples after 7 days of storage, with only minor differences between day 1 and day 14. The total color difference (ΔE*) of lamb meatloaf was calculated depending on the plant additive used. Values above 3.5 indicate a clearly perceptible color difference. In this study, only one such case was observed (ΔE* = 3.96) between the MT and CON samples after 14 days of storage. In the remaining cases, the lowest ΔE* values were recorded for meatloaf with the addition of borage seed oil (BO), ranging from 0.58 to 1.35. For samples with matcha, ΔE* values ranged from 3.11 to 3.33 (BO + MT) and from 1.35 to 3.96 (MT). Importantly, no clear differences were found between the control and experimental samples, which indicates that the product with plant additives does not visually deviate from the reference lamb meatloaf.

### 3.4. Tenderness of Vacuum-Packed Lamb Meatloaf

Comparison of Warner–Bratzler shear force results for meat products revealed a significant (*p* < 0.05) effect of plant-based additives on this parameter (Table 5). Samples with plant additives required less cutting force than the control (CON). Among them, the addition of borage seed oil (BO) resulted in the greatest reduction in sample hardness. A significant (*p* < 0.05) effect of plant additives on decreasing shear force was observed, especially in samples containing BO, throughout the study period.

The addition of BO and/or MT significantly reduced the work required to cut the samples. On days 1 and 7, CON required the greatest cutting work, whereas during the same periods, cutting the BO samples required approximately half the work. After 14 days of storage, the work needed to cut the samples was similar across all treatments. Statistical analysis (*p* < 0.05) confirmed a significant effect of plant additives on reducing cutting work.

## 4. Discussion

### 4.1. Physicochemical Properties of Vacuum-Packed Lamb Meatloaf

Consumption of meat and meat products has beneficial effects on the human body due to the presence of high-quality protein, vitamins, minerals, and other valuable components. However, meat products are perishable and thus require appropriate processing. Thermal treatment is necessary to ensure desirable sensory characteristics, texture, and microbiological safety. This process, however, leads to losses of nutrients and bioactive compounds due to water and volatile compound evaporation, juice leakage, solubilization of components in water, or fat rendering. The extent of these losses depends on the type of meat, as well as the method and duration of processing. Analysis of technological properties, such as cooking yield, allows for the assessment of the impact of different processing methods on meat [39]. Roldan et al. [40] demonstrated that temperature significantly affects the thermal loss of lamb tenderloins, with lower mass loss occurring at 60 °C compared to 80 °C. The baking method used in our study for minced meat (160 °C until reaching an internal temperature of 72 °C) resulted in cooking losses of 32–35%, regardless of the plant-based additive used. The lack of significant differences in cooking yield also corresponded with no differences in moisture content (Table 2). Meanwhile, Kavusan et al. [39] investigated the addition of flaxseed oil emulsion to poultry sausage and observed a decrease in yield after thermal processing, attributing this to a reduced water-binding capacity of proteins in the presence of plant fat. However, Moawad et al. [41], in studies on poultry sausage with green tea extract, reported an increase in yield by 2 percentage points compared to the control (CON). Regarding fat content, it is evident that the addition of oil to the meat filling increased the fat content in samples with BO (Table 2).

The pH value of meat varies depending on the type of raw material, with meat used for sausage production exhibiting a slightly acidic reaction. The acidity level depends not only on the raw material but also on the quality of additional ingredients in the formulation and the storage time of the products [42]. Our study did not show that BO and/or MT affected the pH of vacuum-packed lamb meatloaf stored under refrigeration (Table 2). Similarly, Purnamayanti et al. [43], who added 1–2% green tea leaf powder to lamb sausage stored for 14 days under refrigeration, did not observe a significant effect on pH. However, they noted that in many studies, the pH of meat products decreases when green tea extract is added, mainly due to an increase in lactic acid bacteria [44]. The authors did not observe, when measuring bacterial counts, that green tea powder had the same effect as the extract. Based on this, we also assumed that the addition of MT did not influence the growth of lactic acid bacteria, which could have lowered the pH during storage. Furthermore, plant-based additives with antioxidant and antimicrobial properties, including BO and MT, stabilize the pH of meat products, protecting them from adverse changes [41,45].

### 4.2. Oxidative Stability and Hydrolysis of Vacuum-Packed Lamb Meatloaf

Negative changes in the quality of meat and meat products mainly result from oxidative processes affecting proteins, lipids, and myoglobin autoxidation. These processes lead to the loss of sensory attributes, reduction in nutritional value due to depletion of vitamins and polyunsaturated fatty acids, and the formation of toxic oxidation products [46]. Therefore, monitoring the hydrolytic and oxidative stability of lipids in meat products through the determination of free fatty acids (FFA), TBARS, and peroxide value (PV) is crucial for evaluating meat product quality [42].

Analysis of free fatty acids in meat products allows the assessment of both the rate of oxidative changes and the influence of storage conditions and plant-based additives on lipid stability and product quality. The formation of free fatty acids in meat results from hydrolytic processes, primarily enzymatic lipid degradation [47]. These acids are among the first reagents involved in lipid peroxidation in meat, making their determination an important marker of the rate of oxidative changes in meat products [48]. Literature emphasizes that free fatty acid content increases with prolonged storage of meat products [49]. In the present study, a gradual increase in FFA (by approximately one-third) was observed over 14 days of storage (Table 3), likely due to vacuum packaging of the samples. Mir and Masoodi [50] indicated that vacuum packaging limits the increase in free fatty acids compared to aerobic storage. The level of FFA also depends on the composition of the raw material, particularly the presence of compounds with antioxidant activity. Moreover, spraying chicken breast with an aqueous green tea extract, even during 12 days of aerobic storage, significantly (*p* > 0.05) reduced FFA content compared to the control [49]. Conversely, the introduction of BO, rich in polyunsaturated fatty acids, increased FFA content due to the high susceptibility of oil components to hydrolytic changes.

Peroxide value (PV) determination is a widely used indicator for assessing the degree of oxidative changes in meat products, reflecting the concentration of primary lipid peroxidation products [42]. The rate of lipid peroxidation depends on the chemical structure of fatty acids and the proportion of different fatty acid groups in the meat, with unsaturated fatty acids being the most prone to oxidation. Additionally, the degree of meat fragmentation influences the oxidation rate, as disruption of muscle membrane integrity increases susceptibility to oxidative processes [51]. Authors [49,52] report an increase in peroxide concentration in meat and meat products during storage. The effectiveness of plant-based antioxidants depends on the type of component, level of addition, and chemical properties of the active compounds, such as chemical structure, polarity, and interactions with other ingredients [43]. Kavusan et al. [39] showed that plant oils, such as black cumin and flaxseed oil, exhibit high oxidative instability, resulting in elevated PVs. However, in our study (Table 3), the addition of BO led to a decrease in PV after 7 days of meatloaf storage, which may be attributed to the antioxidant activity of phenolic compounds present in borage seeds. These compounds, present both in aqueous extracts [53] and borage seed oil, can quench reactive oxygen species, limiting oxidation reactions. The reduction and stabilization of PV in MT-containing samples is unsurprising, as matcha tea contains natural antioxidants that delay and limit oxidation through both phenolic compounds and the antioxidant properties of caffeine [54,55] and catechins. These compounds effectively reduce peroxide formation in meat products [45,56,57]. Nain et al. [58] found that green tea extract increased the oxidative stability of oils rich in docosahexaenoic acid (DHA) by reducing the formation of peroxides and secondary oxidation products. Therefore, from a practical standpoint, developing a commercial formulation containing both BO and MT would be advantageous. Storage time also affects PV, as primary oxidation products are unstable and gradually convert into secondary lipid or protein peroxidation products [59]. The variability of PV during storage highlights the importance of monitoring oxidative stability to maintain meat quality.

TBARS analysis is a widely used tool to assess lipid oxidation in meat products. This test measures malondialdehyde (MDA), a secondary lipid peroxidation product, expressed in mg MDA/kg product [48]. Low MDA content indicates initial signs of meat rancidity, whereas exceeding 2 mg MDA/kg product may result in undesirable sensory changes [26]. The value of this indicator largely depends on thermal processing—high temperatures, such as grilling, significantly increase MDA concentration compared to cooking at lower temperatures due to intensified lipid oxidation [60]. Technological factors, such as meat grinding and the addition of curing salt, can also accelerate MDA formation [61]. However, plant-based additives significantly limit lipid oxidation. Our study shows that the addition of BO reduced MDA levels in the product compared to CON (Table 5). Other studies [39,62] indicate that plant oils rich in unsaturated fatty acids lower TBARS values, despite the susceptibility of these fatty acids to oxidation. As far as we know, BO has not been previously used in studies on lipid oxidation in meat products. Nevertheless, the literature reports that aqueous extracts of borage seeds reduce lipid oxidation in various meat and fish products [11,13,15,16]. The addition of MT reduced MDA content by more than 70% compared to CON (Table 3). Green tea is rich in polyphenols [49]. The antioxidant activity of phenolic compounds is closely related to the hydroxyl group attached to the aromatic ring, which can donate electrons with hydrogen atoms to neutralize free radicals. This mechanism blocks further degradation of highly reactive oxidizing species, preventing the formation of lipid oxidation products such as ketones and aldehydes. As a result, low MDA levels are observed in the final product, as also reported in other studies [11,43,49]. Storage time of meat products also affects TBARS values [39,57,59]. With refrigerated storage extended to 14 days, an increase in MDA was observed in CON and, to a lesser extent, in the BO trial, indicating intensified lipid oxidation. The highest MDA values after 14 days of storage were 2.27 and 1.80 mg/kg product, respectively. In samples with MT, MDA levels remained low and stable. The presence of plant antioxidants effectively limits the rate of oxidative changes occurring during refrigerated storage [46].

### 4.3. Color Parameters CIE L*a*b* of Vacuum-Packed Lamb Meatloaf

The color of meat and meat products is one of the most important quality indicators. Subjective color evaluation influences the perception of tenderness, palatability, and freshness of the product. Meat color depends on the content and form of heme pigments, the rate of myoglobin transformations in contact with oxygen, the physicochemical properties of muscle tissue, as well as water and fat content [61]. Additionally, color is influenced by the formulation, meat proportion, amount of added water, and the presence of plant-based ingredients. Color values also depend on the thermal processing method, processing temperature, and storage duration [63]. Higher temperatures above 70 °C accelerate protein denaturation and aggregation, resulting in more intense light scattering and reduced meat lightness [39]. The conversion of myoglobin to brown-colored forms can be enhanced by the presence of salts, which exhibit pro-oxidative activity by detaching iron atoms from heme pigment molecules [64].

The physicochemical properties of plant-based additives determine the hue of meat products—dark and intensely colored components cause greater changes in color parameters than lighter components. Measurement of color parameters (Table 5) indicated that the addition of BO did not significantly affect the lightness of lamb products. Meanwhile, other authors [62,65] reported an increase in L* values with higher oil levels. This increase in lightness was attributed to the better dispersion of plant oil compared to animal fat, allowing incident light to scatter more effectively, thus enhancing the lightness effect. Furthermore, smaller oil droplets have a larger surface area, which may lead to greater light reflection. Our results may not have confirmed these observations due to the low level of added oil, whereas other studies used 5–30% oil. In contrast, powdered green tea imparts a darker color to products, as confirmed by Bellés et al. [11], who stored raw lamb legs sprayed with aqueous extracts of matcha or borage for 13 days, and Jamróz et al. [66], who used green tea extracts in edible films prepared from furcellaran-gelatin blends. Wereńska-Sudnik et al. [59] and Bellés et al. [11] demonstrated that product lightness tends to decrease during storage. Our study, similarly to Uzlasir et al. [62], who used pumpkin seed oil as a partial animal fat replacer in Bologna sausages, did not confirm these observations.

Samples with the addition of MT were characterized by significantly lower redness values (a*) compared to CON (*p* < 0.05) (Table 5). Chlorophyll and carotenoids present in tea extracts impart a yellow-green pigmentation to the products. The reduced redness observed with the addition of oil, also reported by Uzlasir et al. [62] and Ambrosiadis et al. [67], is likely due to the distribution of the oil phase within the myofibrillar protein matrix, which increases the surface area of fat droplets. They noted that the inclusion of plant-based components, such as green tea or pumpkin seed oil, results in meat discoloration, shifting the hue toward green tones. Red color saturation also decreased over storage time, regardless of the additives used. The chromaticity of the yellow–blue axis (b*) is influenced by the formulation and storage duration. No significant effect of adding 4% BO and/or 0.5% MT on the b* parameter was observed. However, other studies [62,68] demonstrated that higher oil levels (>5%) increased yellowness (b*), likely due to the yellow color of the oil [69]. Literature reports also confirm the effect of green tea addition on meat product yellowness. However, Akcan et al. [70] and Jamróz et al. [66] emphasized that this was a result of acidic pH acting on chlorophyll in matcha tea, which degrades to pheophytin and pheophorbide. Consequently, this intensifies olive-green or yellow tones, leading to increased yellowness in samples with green tea addition. Nevertheless, color is a key quality attribute of lamb meat valued by consumers at the point of purchase. Therefore, the protective effect stabilizing color parameters, provided by the proposed addition of 4% BO and 0.5% MT, may positively influence consumer purchasing decisions.

### 4.4. Tenderness of Vacuum-Packed Lamb Meatloaf

Tenderness along with flavor and aroma, is one of the key indicators of meat quality. It is defined as the ease with which a bite can be bitten off, chewed, and swallowed. Tenderness is inversely related to the instrumentally measured maximum shear force. According to Novaković and Tomasević [71], this method provides a better overall assessment of product texture than the TPA (Texture Profile Analysis) test. Among the quality attributes of meat products, tenderness is the least stable. It depends on multiple factors, including the structure of muscle fibers, especially myofibrils, and the content of connective tissue, which varies depending on the animal species, age, sex, and feeding system [72]. Postmortem processes and meat aging also have a significant impact [73]. Additionally, tenderness is influenced by the method of thermal processing, temperature, and duration [47]. During thermal processing, collagen dissolves, increasing tenderness, whereas myofibrillar proteins denature, causing meat contraction and increased firmness [74]. Processing at temperatures above 70 °C reduces meat tenderness [63].

The addition of borage seed oil, or the combination of borage seed oil and matcha, significantly increased the tenderness of lamb meatloaf. Samples containing only matcha and the control were significantly harder, i.e., less tender (Table 5). Similar effects were observed by Uzlasir et al. [62] when adding plant oil to finely minced beef sausage. They reported that higher pumpkin seed oil content resulted in lower maximum shear force and reduced work of shear. However, Youssef and Barbut [68] showed that the hardness of minced meat products increases with higher protein content. In our study, no modifications were applied to alter protein content. Based on the results of other studies [43,75], it was assumed that protein content was the same across all samples. Tenderness is also affected by moisture content [76]. However, our results indicate that the addition of BO and/or MT did not significantly change the moisture content. Similarly, Purnamayanti et al. [43], who used 1–2% powdered green tea in lamb sausage, observed no effect on moisture content or tenderness. Likewise, Cegiełka [77] and Serdaroglu et al. [78] did not report significant differences in moisture content in meat products with added plant oils. In the authors’ opinion, the increased tenderness of samples containing plant oils can be attributed to their physicochemical properties. Plant fats have a significantly lower melting point than animal fats due to their higher content of unsaturated fatty acids. As a result, when incorporated into comminuted meat products, these oils form a softer and less cohesive matrix at room or refrigerated temperatures, contributing to a more tender texture compared to products containing primarily animal fats, which are richer in saturated fatty acids and solidify more readily. Additionally, the presence of these oils may interact with water and protein components, further influencing the product’s structural characteristics and overall mouthfeel. Moreover, the addition of matcha powder, due to its hygroscopic properties, may also enhance in the absorption and retention of water during storage, which affects the texture and juiciness of the product.

### 4.5. Statistical Analysis-Principal Component Analysis

**Figure 1:**
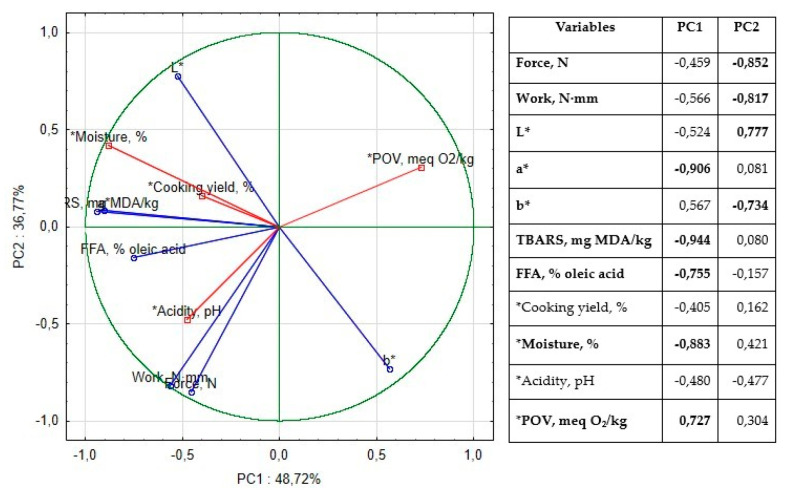
Loadings for the two principal components and table of correlations of variables to factors in principal component analysis based on factor loadings. Correlations above 0.700 were marked in bold.

To evaluate the impact of borage seed oil (BO) and matcha tea (MT) on the physicochemical properties, oxidative stability, color, and tenderness of vacuum-packed lamb meatloaf during 14 days of storage at 4 °C, Principal Component Analysis (PCA) was conducted. The first two principal components accounted for 85.49% of the total variance (Figure 1).

The first component (PC1) explained 48.77% of the variability, with TBARS, redness (a*), moisture, and FFA showing strong negative correlations, while peroxide value (PV) exhibited a strong positive correlation. The most influential contributions to PC1 were from the CON sample (64.87%) and the BO + MT sample (12.52%). The second component (PC2), explaining 36.77% of the variance, was positively associated with lightness (L*) and negatively associated with yellowness (b*) and tenderness. The BO sample had the highest contribution to PC2 (89.49%). These results suggest that the combined use of BO and MT influences oxidative stability, color parameters, and texture, highlighting their potential synergistic effects in lamb meatloaf during refrigerated storage.

## 5. Conclusions

The results of this study indicate a beneficial combination of plant oils and powdered green tea in meat products, suggesting a promising direction for further research aimed at improving the quality and stability of processed meat. Lamb, as a high-quality raw material, possesses a favorable nutritional profile and delicate flavor, making it an excellent base for the development of innovative meat products enriched with natural plant-based additives. The innovative combination of plant-derived additives, such as borage seed oil and matcha tea, in meat products ensures high oxidative stability. The synergistic action of phenolic compounds and oil components limits oxidation processes, enhancing product quality and safety. Instrumental measurement of color parameters showed no significant differences in color between control and experimental samples, while the addition of oil increased the tenderness of the lamb meatloaf. These findings may be relevant for the food industry, as they suggest that incorporating natural plant antioxidants into meat formulations can improve product stability and quality, meet consumer demand for clean-label foods, and open new opportunities for developing functional meat products with extended shelf life and additional health benefits. Further research should focus on in-depth analysis of bioactive properties and consumer studies.

## Figures and Tables

**Table 1:** Samples of lamb meatloaf.

Samples	Lamb Meat [%]	Salt [%]	Water [%]	Borage Seed Oil [%]	Matcha Tea Powder [%]
BO + MT	100	2	10	4	0.5
BO	100	2	10	4	-
MT	100	2	10	-	0.5
CON	100	2	10	-	-

Explanatory notes: BO + MT—lamb meatloaf with 4% borage seed oil and 0.5% matcha tea powder; BO—lamb meatloaf with 4% borage seed oil; MT—lamb meatloaf with 0.5% matcha tea powder; CON—lamb meatloaf without plant additives.

**Table 2:** Physicochemical properties of vacuum-packed lamb meatloaf (mean ± standard deviation).

	Storage Time [Days]	Cooking Yield [%]	Moisture [%]	Fat Content [%]	pH [- ]
BO + MT	1	65.63 ± 1.32 ^a^	53.95 ± 1.48 ^a^	15.54 ± 0.85 ^b^	5.92 ± 0.07 ^a^
	7				5.95 ± 0.05 ^a^
	14				5.97 ± 0.05 ^a^
BO	1	65.04 ± 3.09 ^a^	55.34 ± 2.29 ^a^	15.44 ± 0.72 ^b^	5.92 ± 0.06 ^a^
	7				5.95 ± 0.07 ^a^
	14				5.96 ± 0.06 ^a^
MT	1	67.20 ± 2.87 ^a^	53.45 ± 2.17 ^a^	11.63 ± 0.23 ^a^	5.93 ± 0.06 ^a^
	7				5.98 ± 0.04 ^a^
	14				5.98 ± 0.04 ^a^
CON	1	67.54 ± 2.76 ^a^	56.80 ± 2.41 ^a^	11.42 ± 0.11 ^a^	5.93 ± 0.06 ^a^
	7				5.98 ± 0.01 ^a^
	14				5.98 ± 0.02 ^a^

Explanatory notes: BO + MT—lamb meatloaf with 4% borage seed oil and 0.5% matcha tea powder; BO—lamb meatloaf with 4% borage seed oil; MT—lamb meatloaf with 0.5% matcha tea powder; CON—lamb meatloaf without plant additives. Samples marked with the same letter in the columns are not significantly different (*p* > 0.05).

**Table 3:** Oxidative stability and hydrolysis of vacuum-packed lamb meatloaf (mean ± standard deviation).

	Storage Time [Days ]	FFA [% Oleic Acid]	PV [meqO_2_/kg]	TBARS [MDA/kg]
BO + MT	1	1.96 ± 0.13 ^f^	6.86 ± 0.19 ^c^	0.64 ± 0.06 ^e^
	7	2.45 ± 0.12 ^cd^	6.44 ± 0.17 ^e^	0.74 ± 0.13 ^e^
	14	2.61 ± 0.19 ^bc^	8.26 ± 0.23 ^a^	0.69 ± 0.16 ^e^
BO	1	1.88 ± 0.15 ^f^	6.99 ± 0.19 ^cd^	1.12 ± 0.11 ^d^
	7	2.31 ± 0.18 ^de^	6.19 ± 0.14 ^f^	1.36 ± 0.13 ^c^
	14	2.43 ± 0.17 ^cd^	8.30 ± 0.22 ^a^	1.80 ± 0.08 ^b^
MT	1	1.46 ± 0.07 ^f^	6.77 ± 0.17 ^ad^	0.47 ± 0.03 ^f^
	7	2.06 ± 0.16 ^e^	6.56 ± 0.11 ^e^	0.54 ± 0.14 ^ef^
	14	2.76 ± 0.20 ^ab^	8.04 ± 0.18 ^a^	0.48 ± 0.06 ^f^
CON	1	1.81 ± 0.11 ^f^	7.14 ± 0.16 ^bc^	1.66 ± 0.15 ^c^
	7	2.18 ± 0.16 ^e^	7.32 ± 0.18 ^b^	1.80 ± 0.12 ^b^
	14	2.84 ± 0.14 ^a^	7.34 ± 0.15 ^b^	2.27 ± 0.18 ^a^

Explanatory notes: BO + MT—lamb meatloaf with 4% borage seed oil and 0.5% matcha tea powder; BO—lamb meatloaf with 4% borage seed oil; MT—lamb meatloaf with 0.5% matcha tea powder; CON—lamb meatloaf without plant additives; FFA—free fatty acids; TBARS—thiobarbituric acid reactive substances (TBARS); PV—peroxide value. Samples marked with the same letter in the columns are not significantly different (*p* > 0.05).

**Table 4:** Color parameters CIE L*a*b* of vacuum-packed lamb meatloaf (mean ± standard deviation).

	Storage Time [Days]	L* [-]	a* [-]	b* [-]	ΔE*
BO + MT	1	54.38 ± 2.21 ^a^	1.97 ± 0.22 ^d^	12.18 ± 0.80 ^a^	3.11
	7	53.74 ± 2.15 ^a^	2.03 ± 0.15 ^d^	12.58 ± 0.53 ^a^	2.67
	14	55.55 ± 1.55 ^a^	1.91 ± 0.24 ^d^	12.13 ± 0.42 ^a^	3.33
BO	1	56.92 ± 1.39 ^a^	4.39 ± 0.25 ^ab^	11.76 ± 0.77 ^a^	1.11
	7	54.73 ± 1.65 ^a^	3.98 ± 0.32 ^b^	12.06 ± 0.69 ^a^	0.58
	14	57.75 ± 1.63 ^a^	3.16 ± 0.13 ^c^	11.76 ± 0.57 ^a^	1.35
MT	1	54.18 ± 2.07 ^a^	1.87 ± 0.23 ^de^	11.66 ± 0.48 ^a^	1.35
	7	52.60 ± 1.84 ^a^	1.85 ± 0.18 ^de^	12.02 ± 0.60 ^a^	3.22
	14	55.07 ± 1.50 ^a^	1.69 ± 0.25 ^e^	10.97 ± 0.34 ^b^	3.96
CON	1	55.87 ± 1.87 ^a^	4.61 ± 0.26 ^a^	11.49 ± 0.47 ^ab^	
	7	54.62 ± 1.46 ^a^	4.38 ± 0.44 ^ab^	11.66 ± 0.48 ^ab^	
	14	57.67 ± 1.91 ^a^	4.11 ± 0.18 ^b^	10.80 ± 0.52 ^b^	

Explanatory notes: BO + MT—lamb meatloaf with 4% borage seed oil and 0.5% matcha tea powder; BO—lamb meatloaf with 4% borage seed oil; MT—lamb meatloaf with 0.5% matcha tea powder; CON—lamb meatloaf without plant additives; L*—lightness; a*—redness; b*—yellowness; ΔE*—total difference in color. Samples marked with the same letter in the columns are not significantly different (*p* > 0.05).

**Table 5:** Tenderness of vacuum-packed lamb meatloaf (mean ± standard deviation).

	Storage Time [Days]	Maximum Shear Force [N]	Work of Shear [N·mm]
BO + MT	1	4.70 ± 0.45 ^e^	57.16 ± 8.78 ^c^
	7	6.11 ± 0.60 ^bd^	71.35 ± 7.46 ^b^
	14	5.36 ± 0.80 ^d^	63.53 ± 5.78 ^bc^
BO	1	5.09 ± 0.24 ^e^	53.32 ± 7.48 ^c^
	7	5.42 ± 0.47 ^de^	66.10 ± 5.93 ^b^
	14	4.91 ± 0.33 ^e^	63.81 ± 6.90 ^bc^
MT	1	6.51 ± 0.74 ^abc^	76.44 ± 6.05 ^b^
	7	6.22 ± 0.50 ^bc^	71.62 ± 7.14 ^b^
	14	6.19 ± 0.43 ^c^	71.21 ± 6.96 ^b^
CON	1	7.19 ± 0.52 ^ab^	100.28 ± 11.90 ^a^
	7	7.22 ± 0.04 ^a^	113.80 ± 13.23 ^a^
	14	5.70 ± 0.53 ^d^	68.89 ± 6.60 ^b^

Explanatory notes: BO + MT—lamb meatloaf with 4% borage seed oil and 0.5% matcha tea powder; BO—lamb meatloaf with 4% borage seed oil; MT—lamb meatloaf with 0.5% matcha tea powder; CON—lamb meatloaf without plant additives. Samples marked with the same letter in the columns are not significantly different (*p* > 0.05).

## Data Availability

The original contributions presented in this study are included in this article. Further inquiries can be directed to the corresponding author.

## Abbreviations

The following abbreviations are used in this manuscript:

BO + MT	lamb meatloaf with 4% borage seed oil and 0.5% matcha tea powder
BHT	butylated hydroxytoluene
BO	lamb meatloaf with 4% borage seed oil
CLA	conjugated linoleic acid
CON	lamb meatloaf without plant additives
FFA	free fatty acids
MT	lamb meatloaf with 0.5% matcha tea powder
PCA	principal component analysis
PV	peroxide value
PUFA	polyunsaturated fatty acids
TBA	2-thiobarbituric acid
TBARS	thiobarbituric acid reactive substances
TPA	texture profile analysis
